# Mitsugumin 29 regulates t-tubule architecture in the failing heart

**DOI:** 10.1038/s41598-017-05284-2

**Published:** 2017-07-13

**Authors:** Robert N. Correll, Jeffrey M. Lynch, Tobias G. Schips, Vikram Prasad, Allen J. York, Michelle A. Sargent, Didier X. P. Brochet, Jianjie Ma, Jeffery D. Molkentin

**Affiliations:** 1 Department of Pediatrics, University of Cincinnati, Cincinnati Children’s Hospital Medical Center, Cincinnati, Ohio 45229 USA; 2 Department of Physiology, University of Maryland School of Medicine and Center for Biomedical Engineering and Technology (BioMET), Baltimore, Maryland 21201 USA; 3 Department of Surgery, The Ohio State University, Columbus, Ohio 43210 USA; 4 Howard Hughes Medical Institute, Cincinnati, Ohio 45229 USA

## Abstract

Transverse tubules (t-tubules) are uniquely-adapted membrane invaginations in cardiac myocytes that facilitate the synchronous release of Ca^2+^ from internal stores and subsequent myofilament contraction, although these structures become disorganized and rarefied in heart failure. We previously observed that mitsugumin 29 (Mg29), an important t-tubule organizing protein in skeletal muscle, was induced in the mouse heart for the first time during dilated cardiomyopathy with heart failure. Here we generated cardiac-specific transgenic mice expressing Mg29 to model this observed induction in the failing heart. Interestingly, expression of Mg29 in the hearts of *Csrp3* null mice (encoding muscle LIM protein, MLP) partially restored t-tubule structure and preserved cardiac function as measured by invasive hemodynamics, without altering Ca^2+^ spark frequency. Conversely, gene-deleted mice lacking both Mg29 and MLP protein showed a further reduction in t-tubule organization and accelerated heart failure. Thus, induction of Mg29 in the failing heart is a compensatory response that directly counteracts the well-characterized loss of t-tubule complexity and reduced expression of anchoring proteins such as junctophilin-2 (Jph2) that normally occur in this disease. Moreover, preservation of t-tubule structure by Mg29 induction significantly increases the function of the failing heart.

## Introduction

Cardiac contraction is initiated by membrane depolarization and Ca^2+^ release from voltage-gated L-type Ca^2+^ channels (LTCC) within the sarcolemma and transverse tubules (t-tubules). This depolarization and Ca^2+^ release directly stimulates apposing type 2 ryanodine receptors (RyR2) within the sarcoplasmic reticulum (SR), thereby releasing a large pool of Ca^2+^ for contraction. Due to the large size of cardiac myocytes the majority of LTCCs (approximately 80%)^1^ are localized within t-tubules, which are specialized sarcolemmal invaginations that allow synchronous activation of RyR2 and coordinated myofilament contraction throughout the entirety of the cell^2, 3^. Cardiac t-tubules are dynamic structures that form during development^4^ in a pattern that overlies the sarcomeric z-lines. In select mouse models of heart disease, t-tubule remodeling occurs that is characterized by their fragmentation and rarefaction, which correlates with desynchronized Ca^2+^ coupling between the LTCCs and RyR2, as well as reduced contractile function^2^. Recent data from human patients has demonstrated that alterations in t-tubule organization are associated with loss of contractile function^2, 5^. This reduced t-tubule organization is thought to occur primarily via loss of structural proteins such as junctophilin-2 (Jph2) that are required for their anchoring^6, 7^.

T-tubules in both cardiac and skeletal muscle myocytes are thought to form in a caveolin-dependent manner^8, 9^ but the morphology of the t-tubules and the structural proteins that organize them differ. Mitsugumin 29 (Mg29, encoded by the *Sypl2* gene) is a structural protein that is thought to be exclusively expressed in skeletal muscle where it is localizes to both t-tubules and the SR terminal cisternae^10, 11^. Skeletal muscles from *Sypl2* gene-deleted mice display swollen and disorganized t-tubules or lack t-tubules entirely^12, 13^. Functionally, Mg29 interacts with transient receptor potential canonical 3 (TRPC3)^14, 15^ and *Sypl2* deletion results in the complete loss of store-operated Ca^2+^ entry in skeletal muscle^16^ that results in increased fatigability^17, 18^. Mg29 mRNA is not appreciably expressed in the mouse heart, and the protein is undetectable by immunoblotting^10^. However, Mg29 mRNA is lowly expressed in the human heart^19^, and Mg29 was recently shown to be upregulated at both the mRNA and protein level in a guinea pig model of heart failure^20^.

Here we show that Mg29 mRNA is upregulated in multiple mouse models of heart failure. To model this increase we created transgenic mice with cardiac-restricted expression of Mg29 protein, which was localized to t-tubules adjacent to RyR2 in the apposed SR. When crossed into the well-characterized *Csrp3* ^−/−^ mouse model of dilated cardiomyopathy, the Mg29 transgene was protective, increasing t-tubule organization and preserving both cardiac contraction and relaxation. Double *Sypl2* ^−/−^ *Csrp3* ^−/−^ mice showed a further disorganization of t-tubules and even worse cardiac function compared with *Csrp3* ^−/−^ mice alone. These data suggest that induction of Mg29 in the diseased heart serves a compensatory role in maintaining t-tubule organization and preserving cardiac function.

## Results

### Mg29 expression characteristics in the mouse heart

**Figure 1:**
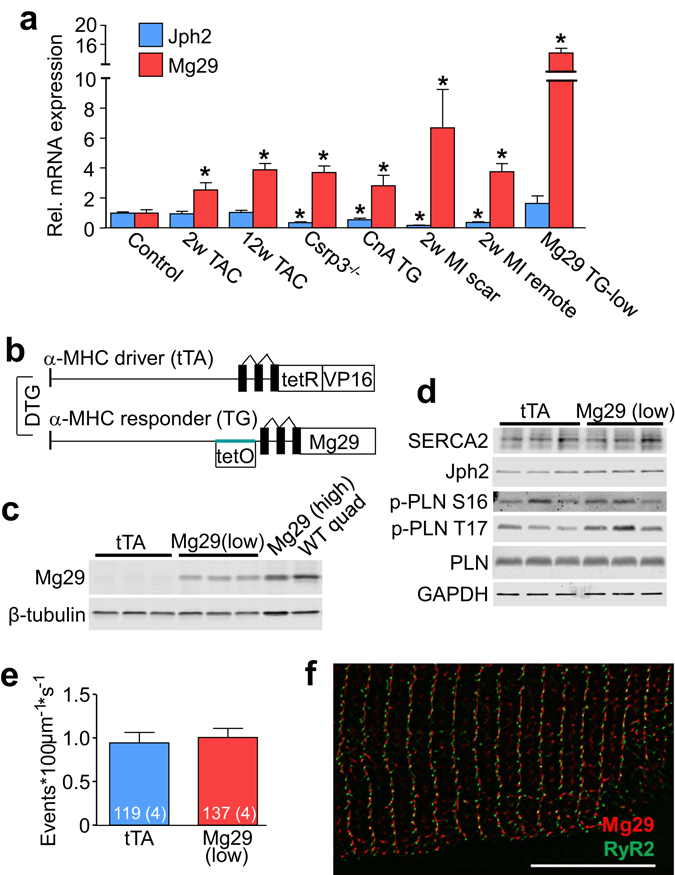
Mg29 mRNA is induced during heart failure. (**a**) Quantitative PCR demonstrating increased expression of Mg29 mRNA (red) and reduced expression of Jph2 mRNA (blue) in hearts of both *Csrp3* ^−/−^ mice lacking MLP and calcineurin A (CnA) transgenic mice, as well as both the scar and remote regions of hearts after two weeks of myocardial infarction (MI). Hearts subjected to either 2 or 12 weeks of transverse aortic constriction (TAC) surgery showed induction of Mg29 but no change in Jph2 levels. Mg29 Low transgenic hearts (described below) showed approximately 14-fold induction of Mg29 message compared to WT control hearts. Hearts from 6 control mice, 6 two-week TAC mice, 10 twelve-week TAC mice, 5 *Csrp3* ^−/−^ mice, 6 CnA transgenic mice, 5 two-week MI mice, and 4 Mg29 Low transgenic mice were utilized in this experiment. *P < 0.05 compared to expression in control mice. (**b**) Schematic representation of double transgenic (DTG) system used to express Mg29 in the mouse heart. (**c**) Immunoblot demonstrating expression of Mg29 protein in the DTG mouse heart (Mg29 High) at comparable levels to that seen in wild-type quadriceps muscle (WT Quad). Administration of DOX in the chow reduced Mg29 levels (Mg29 Low). Cropped western blots images are shown although the full-length gel files can be viewed in Supplemental Fig. 3. Photoshop was used to process the images, although they were not manipulated to alter the data. (**d**) Immunoblots of Ca^2+^ handling proteins in DTG hearts expressing Mg29 or tTA-expressing control hearts. Phospholamban (PLN) and its phosphorylated forms at S16 and T17 were also unchanged. Cropped western blots images are shown although the full-length gel files can be viewed in Supplemental Fig. 3. Photoshop was used to process the images, although they were not manipulated to alter the data. (**e**) Ca^2+^ spark frequency measured in myocytes isolated from DTG hearts expressing Mg29 or tTA-expressing control hearts. Number of myocytes and animals used are shown in the graph. (**f**) Super-resolution confocal microscopy of an isolated adult cardiomyocyte from a DTG heart showing that Mg29 (red) and RyR2 (green) are adjacent but not overlapping. Scale bar is 10 µm.

We previously identified that high-expressing myocyte enhancer factor-2A (MEF2A) cardiac-specific transgenic mice develop heart failure by 3 weeks of age and showed induction of Mg29 mRNA expression^21^, a known skeletal muscle-specific gene^10^. We subsequently identified Mg29 mRNA as induced in the hearts of mice following transverse aortic constriction (TAC), myocardial infarction (MI) injury, in transgenic mice expressing activated calcineurin (CnA), and strongly in *Csrp3* ^−/−^ mice lacking muscle LIM protein (MLP), which is a model of dilated cardiomyopathy (Fig. 1a)^22^. In keeping with the well-characterized loss of t-tubule structure during heart failure, we identified that mRNA for junctophilin-2 (Jph2), which is a major cardiac t-tubule anchoring and structural protein^6, 7^, was significantly downregulated in mouse hearts lacking *Csrp3* ^−/−^, as well as hearts from mice overexpressing CnA, and in both scar and remote regions of mouse hearts 2 weeks after MI surgery. Notably, however, pressure overload induced by either 2 or 12 weeks of pressure overload induced by transverse aortic constriction (TAC) surgery had no effect on Jph2 mRNA expression in the mouse heart (Fig. 1a).

To model this induction of Mg29 expression in the failing mouse heart we utilized a cardiac-specific binary transgenic system in which the presence of both a tetracycline-transactivator (tTA) protein under the control of the α-myosin heavy chain (α-MHC) promoter and a Mg29 responder transgene together produces overexpression of the Mg29 protein in the absence of doxycycline (DOX), as described previously (Fig. 1b)^23^. Hearts from double transgenic (DTG) mice showed high levels of Mg29 expression similar to native levels in the quadriceps muscle (Fig. 1c). However, this high line developed heart failure on its own so it was not used. DTG mice on DOX chow that had “leak” expression were used for most subsequent experiments because this lower level of expression was still robust for the heart, showing approximately 14-fold greater expression of Mg29 message in the heart compared with WT controls (Fig. 1a) although it was completely innocuous at 5 months of age (as will be discussed later).

Low-level expression of Mg29 resulted in a small but significant increase in Jph2 protein levels, but had no effect on the expression of other Ca^2+^ handling proteins localized to the SR or t-tubules (Fig. 1d and Fig. S1). Since Mg29 has been described as both a t-tubule and SR-resident protein in skeletal muscle^11^, and it was previously identified as a potential regulator of RyR1 activity^24^, we measured Ca^2+^ spark frequency and found no effect with Mg29 expression (Fig. 1e). To extend these findings, super-resolution confocal imaging was employed using the recently-described super-resolution radial fluctuations (SRRF) technique^25^ to examine the localization of Mg29 and RyR2 in isolated cardiomyocytes from DTG mice. We observed that Mg29 was localized adjacent to RyR2 but not coincident with it (Fig. 1f). These data indicate that in the heart, Mg29 is localized to t-tubules, not the SR, and that low-level expression of Mg29 does not seem to influence RyR2 activity directly.

### Mg29 expression partially preserves t-tubule structure and cardiac function in *Csrp3*^−/−^ mice

**Figure 2:**
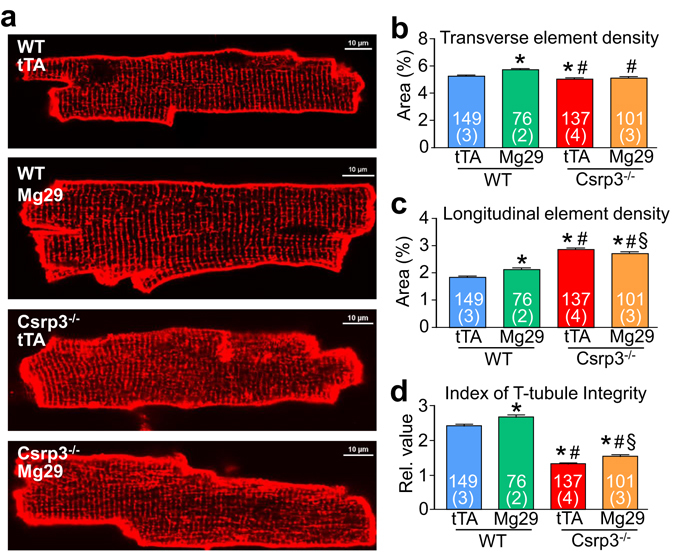
Transgenic expression of Mg29 in hearts of mice lacking MLP protein results in improved t-tubule organization. (**a**) Confocal images of di-8-ANEPPS-loaded cardiac myocytes isolated from wild-type (WT) mice expressing Mg29 or tTA (control), and *Csrp3* ^−/−^ mice expressing Mg29 or tTA. Densities of cardiac myocyte transverse t-tubule elements (**b**), longitudinal t-tubule elements (**c**), and combined measures of t-tubule integrity (**d**) were generated using AutoTT software. Number of cardiomyocytes (upper number) and mice analyzed (lower number) is shown in the graphs. *P < 0.05 versus tTA (control) group. ^#^P < 0.05 versus Mg29 DTG group. ^§^P < 0.05 versus *Csrp3* ^−/−^, tTA group.

**Figure 3:**
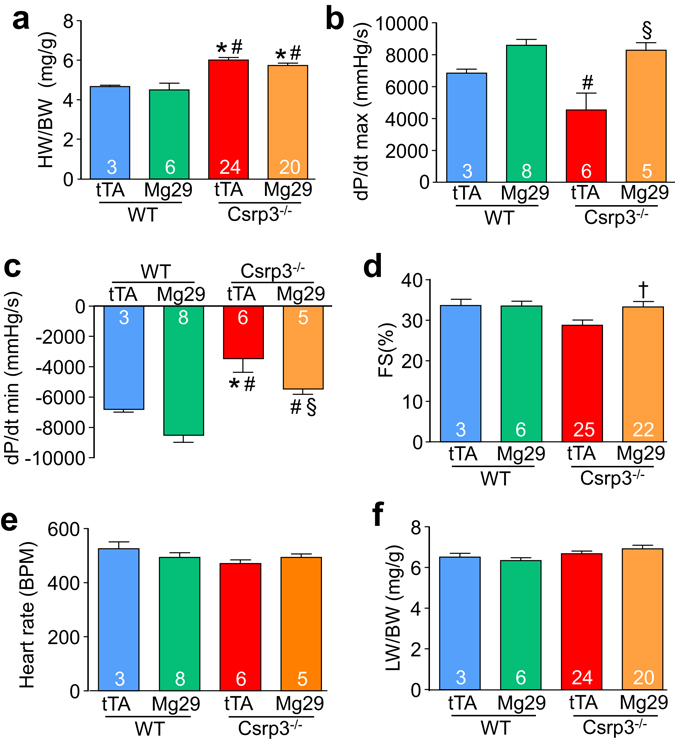
Transgenic expression of Mg29 improves cardiac functional performance in mice lacking MLP protein. (**a**) Heart weight normalized to body weight (HW/BW), (**b**) systolic function (dP/dt max), and (**c**) relaxation (dP/dt min), (**d**) fractional shortening (FS) percentage, (**e**) heart rate in beats per minute (BPM), and (**f**) lung weight normalized to body weight (LW/BW) measured from low-expressing Mg29 DTG mice, tTA-expressing control mice, and *Csrp3* ^−/−^ mice expressing either Mg29 or tTA. Number of mice analyzed is shown in the bars of each graph. *P < 0.05 versus tTA (control) group. ^#^P < 0.05 versus Mg29 DTG group. ^§^P < 0.05 versus *Csrp3* ^−/−^, tTA group. ^†^P < 0.05 versus tTA *Csrp3* ^−/−^ group by Student’s t-test, although this difference was not quite significant by ANOVA.

Given that Mg29 was previously described as an important regulator of t-tubule structure in skeletal muscle^12, 13^, and that *Csrp3* ^−/−^ mice develop heart failure^22^ that is associated with reduced t-tubule architecture, we crossed Mg29 DTG mice into the *Csrp3* ^−/−^ background. At 12–15 weeks of age, *Csrp3* ^−/−^ mice expressing Mg29 had significantly improved t-tubule integrity compared to control *Csrp3* ^−/−^ mice expressing only the single tTA transgene (Fig. 2a,d), including a reduction in longitudinal t-tubule elements that is a hallmark of heart failure (Fig. 2a,c). Surprisingly, wildtype Mg29 DTG mice alone showed increased t-tubule integrity at baseline (Fig. 2a,d) reflecting an increase in total t-tubule density (Fig. 2a–c) but not regularity. Although the increase in t-tubule density and organization observed here was subtle, it resulted in a more substantial and significant restoration in systolic function in the *Csrp3* ^−/−^ background at 15 weeks of age (Fig. 3b), as well as significantly improved relaxation (Fig. 3c) with no change in resting heart rate (Fig. 3e). *Csrp3* ^−/−^ mice expressing Mg29 also demonstrated trends toward reduced heart weight (Fig. 3a) and improved fractional shortening (Fig. 3d, full data in Tables S1, S2) at 5 months of age, perhaps reflecting reduced cardiac remodeling due to the improvement in cardiac function.

### Mg29 expression does not alter cardiac disease progression during pressure overload

**Figure 4:**
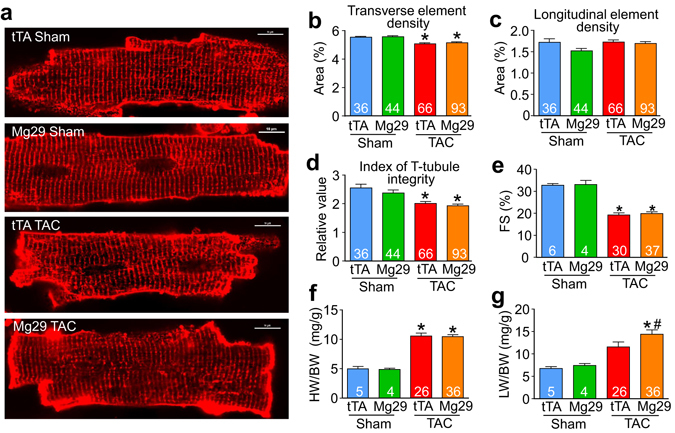
Transgenic expression of Mg29 in the heart does not alter t-tubule morphology or disease progression after long-term pressure overload. (**a**) Confocal images of di-8-ANEPPS-loaded cardiomyocytes isolated from Mg29 Low transgenic or tTA control mice after 12 weeks of TAC or sham control surgery. Densities of cardiomyocyte transverse t-tubule elements (**b**), longitudinal t-tubule elements (**c**), and combined measures of t-tubule integrity (**d**) were generated using AutoTT software. (**e**) Cardiac function as assessed by echocardiographic measures of fractional shortening and (**f**) heart weight normalized to body weight (HW/BW) (**g**) or lung weight normalized to body weight (LW/BW) from Mg29 Low transgenic or tTA control mice after 12 weeks of TAC or sham control surgery. Number of cardiomyocytes (**b**–**d**) and mice analyzed (**e**–**g**) is shown in the bars in each graph. *P < 0.05 versus sham surgery of the same genotype. ^#^P < 0.05 versus tTA of same surgical group.

We next sought to determine whether expression of Mg29 might affect the organization of t-tubules after 12 weeks of pressure overload induced by TAC surgery given that long-term pressure overload induces some aspects of heart failure. However, this stimulus was ultimately different from the type of dilated heart failure observed in *Csrp3* ^−/−^ mice, which have a dramatic downregulation in Jph2 expression, while 12 weeks of TAC in WT mice showed no reduction in Jph2 mRNA expression (Fig. 1a). Indeed, while mice subjected to TAC surgery developed some aspects of heart failure as evidenced by significantly reduced cardiac fractional shortening (Fig. 4e, full data in Tables S3, S4), increased heart weight (Fig. 4f) and pulmonary edema (Fig. 4g), these mice showed relatively little remodeling of t-tubule structure (Fig. 4a) perhaps due to the continued presence of Jph2. Both the density of transversely-oriented tubule elements and the combined index of t-tubule integrity were significantly decreased but not different with or without Mg29 transgene expression (Fig. 4b–d). Consistent with these results, Mg29 expression did not benefit cardiac remodeling/function after TAC as it did in *Csrp3* ^−/−^ mice (Fig. 4e–g). These data suggest a paradigm in which Mg29 expression is only beneficial in a disease context where the native t-tubule organizing protein Jph2 is significantly reduced and t-tubular organization is more dramatically altered.

### Loss of Mg29 further reduces t-tubule organization and cardiac function in *Csrp3*^−/−^ mice

**Figure 5:**
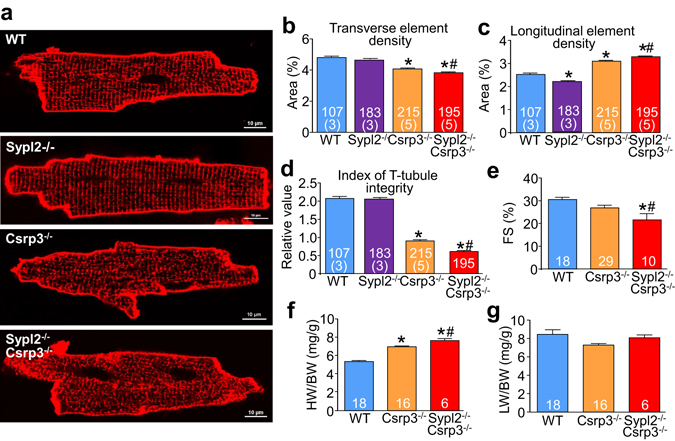
Loss of Mg29 accelerates heart failure progression in *Csrp3* ^−/−^ mice. (**a**) Confocal images of di-8-ANEPPS-loaded cardiomyocytes isolated from *Csrp3* ^−/−^, *Sypl2* ^−/−^, *Csrp3* ^−/−^ *Sypl2* ^−/−^ and WT control mice. Densities of cardiomyocyte transverse t-tubule elements (**b**), longitudinal t-tubule elements (**c**), and combined measures of t-tubule integrity (**d**) were generated using AutoTT software. (**e**) Cardiac function as assessed by echocardiographic measures of fractional shortening, (**f**) heart weight normalized to body weight (HW/BW), and (**g**) lung weight normalized to body weight (LW/BW) from *Csrp3* ^−/−^, *Csrp3* ^−/−^ *Sypl2* ^−/−^ and WT control mice. Number of cardiomyocytes and mice analyzed is shown in the graphs. *P < 0.05 versus WT (control) group. ^#^P < 0.05 versus *Csrp3* ^−/−^ group.

To investigate if the induction of Mg29 that normally occurs in the setting of heart failure due to *Csrp3* deletion was truly compensatory, we crossed *Sypl2* ^−/−^ mice into the *Csrp3* ^−/−^ background and assessed t-tubule organization and cardiac performance. While *Sypl2* ^−/−^ mice lacking Mg29 protein alone showed no significant alterations in t-tubule organization apart from a small decrease in longitudinal element density (Fig. 5c), mice lacking both Mg29 and MLP protein showed significantly reduced t-tubule element density compared with loss of MLP protein alone (Fig. 5a,b). They also showed increased longitudinal t-tubule element density (Fig. 5a,c) and reduced t-tubule integrity (Fig. 5a,d), all of which indicate a worsening of the heart failure phenotype. Consistent with these observations, fractional shortening was reduced even further in the double null mice (Fig. 5e, full data Tables S5, S6) and heart weight normalized to body weight (HW/BW) was significantly increased compared with loss of MLP protein alone (Fig. 5f), although pulmonary edema was not different (Fig. 5g).

To extend these studies, we again sought to determine whether Mg29 induced during long-term pressure overload (Fig. 1a) plays a role in t-tubule organization and cardiac functional performance. To that end, we subjected wild-type or *Sypl2* ^−/−^ mice to 12 weeks of TAC surgery, but found that loss of Mg29 protein had no effect on cardiac remodeling or functional performance apart from a slight reduction in intraventricular septum thickness (Figure S2). Collectively these results suggest that the induction of Mg29 that occurs in mouse models of heart failure where Jph2 expression is lost, such as in *Csrp3* ^−/−^ mice, produces a compensatory response that antagonizes the progression of heart failure through a mechanism involving stabilization of t-tubules. However, in mouse models of heart failure where Jph2 expression is not reduced, augmenting Mg29 expression is of no protective value.

## Discussion

Reduced t-tubule density and organization commonly accompany heart failure in human patients^2, 5^ and in multiple animal models^2^. This rarefaction of t-tubule structure is likely caused by reduced expression or loss of anchoring proteins such as Jph2 that position t-tubules at the z-line^6, 7^, resulting in fragmentation of the t-tubule network. T-tubule fragmentation interrupts coupling between LTCCs and RyR2 in the interior of the myocyte, leading to the dyssynchronous Ca^2+^ release and reduced force generation that typifies heart failure.

Mg29 plays an important role in anchoring t-tubules in skeletal muscle^12, 13^, as muscle fibers from *Sypl2* ^−/−^ mice show swollen, fragmented or even absent t-tubules. While Mg29 is highly expressed in mammalian skeletal muscle we were unable to detect this protein in the mouse heart, suggesting it serves no baseline functional role in this tissue. Indeed, we found no significant difference in gravimetric measures or functional performance of the hearts of *Sypl2* ^−/−^ mice up to 22 weeks of age (Figure S2), nor were t-tubules in cardiomyocytes from these hearts overtly changed compared with wild-type controls (Fig. 5a–d). In the failing mouse heart we observed induction of Mg29 mRNA expression (Fig. 1a)^21^ and both Mg29 mRNA and protein were recently shown to be upregulated in failing guinea pig hearts^20^.

The induction of Mg29 expression observed in *Csrp3* ^−/−^ mice was clearly of functional importance as deletion of the *Sypl2* gene further reduced t-tubule structure and organization and it worsened heart failure in *Csrp3* ^−/−^ mice (Fig. 5). Another interesting aspect of our data is that even a relatively modest improvement in t-tubule structure and organization due to Mg29 induction produced a disproportionately greater rescue of cardiac function in the *Csrp3* ^−/−^ background. Thus, the rarefaction or reorganization of t-tubules that occurs in the failing heart is likely of significant disease relevance.

Because Mg29 also serves as a regulator of store-operated Ca^2+^ entry, possibly via interaction with transient receptor potential canonical 3 (TRPC3)^14–16^, and as an activator of RyR1 activity^24^, we examined the localization of Mg29 using super-resolution confocal imaging^25^. Although it was previously demonstrated by immunogold electron microscopy that Mg29 is localized to both t-tubules and junctional SR in skeletal muscle^11^, in the hearts of our Mg29 DTG mice we observed strict localization only to regions adjacent to but not overlapping with RyR2, suggesting Mg29 is present in t-tubules. Thus, induction of Mg29 expression the failing heart appears to be tailored to regulating t-tubule structure and function, although it certainly could indirectly impact SR Ca^2+^ release by affecting the coordination between these 2 apposed membrane surfaces. Hence, preservation of t-tubule structure should preserve normal excitation-contraction coupling and necessarily improve cardiac function just through this structural mechanism. While we did not measure Ca^2+^ sparks in *Csrp3* ^−/−^ hearts with transgenic overexpression of Mg29, it is possible that Ca^2+^ spark frequency may even be decreased compared with *Csrp3* ^−/−^ alone, as the preserved t-tubule structure found with Mg29 expression should result in fewer “orphaned” RyR2 multimers and the improved coupling could reduce the Ca^2+^ sensitivity required for coordinated activation of RyR2, which may also reduce SR Ca^2+^ leak.

Structurally, Mg29 is composed of a central MARVEL domain that comprises the majority of the protein (75%), flanked by shorter N- and C-terminal cytosolic domains^10, 11^. MARVEL domains are also present in related proteins such as synaptophysin, which is similarly involved in membrane apposition processes and induction of membrane curvature^26^. Since our data demonstrate that Mg29 is localized to t-tubules in the heart it could serve as a structural brace to surround the t-tubule as previously hypothesized^27^. Jph2 is the major t-tubule anchoring protein expressed in the heart^6, 7^, however its expression is frequently reduced in heart failure. Indeed, our results suggest that the effects of Mg29 are only uncovered in heart failure models where Jph2 is downregulated. During long-term pressure overload where Jph2 expression is retained and t-tubules are better preserved, the transgenic expression of Mg29 has no additional effect on t-tubule structure and cardiac disease progression (Fig. 4). Thus, the primary role of Mg29 in the heart appears to be t-tubule stabilization to partially counter the loss of native anchoring proteins such as Jph2 during heart failure. Indeed, we also find that transgenic mice expressing low levels of Mg29 have increased cardiac expression of Jph2 protein (Fig. 1d and Fig. S1), which may further aid Mg29 in preserving t-tubule structure during failure.

Finally, while previous data have shown that Jph2 can stabilize t-tubule structure and cardiac function following pressure overload^28^, it also interacts with and may regulate RyR2 activity^29^, making it difficult to determine whether its role in t-tubule stabilization is solely responsible for the observed improvement in cardiac function. Our results using Mg29, which only affects t-tubules, support the notion that t-tubule stabilization has a critical role in cardiac disease progression through an obvious mechanism of regulating the coupling between the t-tubule and SR membranes to maintain coordinated Ca^2+^ release for contraction. Hence, controlled expression of Mg29 in the failing heart, such as with viral gene therapy approaches, may offer a novel way to correct the reduction in coordinated coupling between the sarcolemma and SR that occurs in heart failure, similar to the strategy recently proposed for Jph2^30^.

## Methods

### Cloning and transgenic mice

A cDNA encoding the *Sypl2* gene was cloned into the murine α-MHC promoter expression vector^23^ and used to inject newly-fertilized oocytes to generate transgenic mice (*FVB/N* background). To test the inducibility of the transgenic system, we administered DOX chow and observed loss of greater than 50% of the Mg29 expression in the heart. Because of lethality we observed in double transgenic (DTG) mice in the absence of DOX, all experiments using transgenic mice were conducted in the presence of DOX to produce low expression of Mg29 protein, with the exception of the experiment shown in Fig. 1F, in which DOX was removed at weaning and mice were aged at least an additional 4 weeks in order to allow for a higher level of protein expression. *Sypl2* null mice^13^ and *Csrp3* null mice^22^ were previously described. For experiments in Fig. 1, mice were in a pure *FVB/N* background. For experiments in Figs 2 and 3, mice are in a mixed *FVB/N* and *C57Bl/6* background. Mice in Fig. 2 were 12–15 weeks of age, mice in Fig. 3a,d,f were 20–22 weeks of age, and mice in Fig. 3b,c,e were 15–17 weeks of age. For pressure overload experiments in Fig. 4 and Figure S2, TAC surgeries were performed on mice at 8–10 weeks of age. For experiments in Fig. 5, mice were in a *C57Bl/6* background. All experiments in Fig. 5 were performed at 5–6 weeks of age (except for *Sypl2* ^−/−^ mice, which were examined at 6–10 weeks of age), as *Csrp3* ^−/−^ mice develop more rapid disease onset in the *C57Bl/6* background in our hands. Both sexes of mice were used and no animals were discarded in the statistical analysis except where noted. Experiments involving animals were approved by the Institutional Animal Care and Use Committee of Cincinnati Children’s and in accordance with the National Institutes of Health Guidelines for the care and use of laboratory animals.

### Western blotting

Hearts were excised from DTG mice expressing the tet-transactivator (tTA) and the inducible promoter for Mg29 expression, in either the presence or absence of DOX, frozen in liquid nitrogen and stored at −80 °C. Ventricles were homogenized in either a modified radioimmunoprecipitation assay (RIPA) buffer containing 1% Triton X-100, 1% sodium deoxycholate, 0.1% SDS, 50 mM Tris-HCl, pH 7.4, and protease inhibitors or a buffer containing 20 mM Tris–HCl, pH 7.5, 250 mM NaCl, 1% Triton X-100, 10 mM MgCl_2_, 0.5 mM dithiothreitol, and protease inhibitors. Homogenates were centrifuged at 14,000 rpm for 10 min and supernatants were used for blotting. Eight to one hundred and twenty micrograms of protein was used, subjected to SDS-PAGE, and transferred to PVDF membranes. Immunoblots were performed using the appropriate primary antibody and fluorescent conjugated secondary antibodies (LI-COR, Lincoln, NE, USA) in combination with an Odyssey CLx Infrared Imaging System (LI-COR). Primary antibodies used were: Mg29 (Sigma-Aldrich, St. Louis, MO, USA), β-tubulin (Developmental Studies Hybridoma Bank, University of Iowa, Iowa City, USA), sarcoplasmic/endoplasmic reticulum Ca^2+^ ATPase 2 (SERCA2, Badrilla, Leeds, United Kingdom), junctophilin-2 (Jph2, Abcam, Cambridge, United Kingdom), phosphorylated phospholamban serine 16 (P-PLN S16, Badrilla, Leeds, United Kingdom), phosphorylated phospholamban threonine 17 (P-PLN T17, Badrilla, Leeds, United Kingdom), phospholamban (PLN, Thermo Fisher Scientific, Waltham, MA, USA), and glyceraldehyde 3-phosphate dehydrogenase (GAPDH, Fitzgerald Industries International, Acton, MA, USA). Western blots were quantified using Image Studio software (LI-COR).

### Ca^2+^ spark measurements

Experiments were performed as previously described^31^. Briefly, isolated adult cardiomyocytes were loaded with 10 µM Fluo-4 (Invitrogen) for 25 min and then placed in Tyrode’s solution containing: 140 mM NaCl, 4 mM KCl, 1.8 mM CaCl_2_, 1 mM MgCl_2_, 10 mM glucose, 5 mM HEPES (pH 7.4). Ca^2+^ sparks were recorded with a Nikon A1 confocal microscope using a GaAsP detector in line scan mode. The cells were stimulated at 0.5 Hz at least 15 times before stimulation was stopped and sparks were recorded at rest. The fluorophore was excited at 488 nm and emission maximum recorded at 525 nm. Sparks were counted using Sparkmaster^32^ and ImageJ (U. S. National Institutes of Health, Bethesda, MD, USA) and calculated as sparks/100 μm/s.

### Immunofluorescence and super-resolution confocal imaging

Cardiac myocytes from adult transgenic mice were isolated as previously described^33^ and imaged with a Nikon A1 confocal microscope using a GaAsP detector. Immunofluorescence was performed using primary antibodies against Mg29 (Sigma-Aldrich, St. Louis, MO, USA) and RyR2 (Abcam, Cambridge, United Kingdom). Super-resolution processing of images was accomplished using super-resolution radial fluctuations (SRRF)^25^ and ImageJ (U. S. National Institutes of Health, Bethesda, MD, USA). For t-tubule imaging experiments, adult cardiac myocytes isolated from mice between 12–15 weeks (Fig. 2a–d), 5 weeks (WT, *Csrp3* ^−/−^, and *Sypl2* ^−/−^ and *Csrp3* ^−/−^ mice in Fig. 5a–d), 6–10 weeks (*Sypl2* ^−/−^ mice in Fig. 5a–d) of age, or after 12 weeks of TAC surgery (Fig. 4a–d) were loaded with 55 µM di-8-ANEPPS for 25 min at room temperature and then placed in a modified Tyrode’s solution containing: 10 mM EGTA, 140 mM LiCl, 4 mM KCl, 1 mM MgCl_2_, 10 mM glucose, 5 mM HEPES (pH 7.4). Images of rod-shaped myocytes were analyzed using AutoTT software^34^.

### Invasive hemodynamics and echocardiography

Hemodynamic measurements were performed as previously described^35^. Briefly, mice were anesthetized by intraperitoneal injection of pentobarbital (6 mg/100 g body weight). A high fidelity, solid state 1.2F pressure catheter (Transonic Scisense Inc, London, ON, Canada) was inserted into the left ventricle (LV) via right carotid exposure and retrograde introduction of the pressure catheter into the LV, and the animal brought to 37 °C. Data were collected by PowerLab 8/36 (ADInstruments, Sydney, Australia) and recorded using LabChart 7 Pro (ADInstruments, Sydney, Australia). Ten seconds of recorded data was averaged for each time point. Echocardiography was performed on mice after isoflurane inhalation for anesthesia (dosage was to effect) using a Hewlett Packard 5500 instrument with a 15-MHz microprobe and measurements were taken on M-mode in triplicate for each mouse and averaged. Analgesia was not used in these experiments because echocardiography is non-invasive, and hemodynamic assessment was a terminal procedure.

### Pressure overload and myocardial infarction surgeries

Eight to ten week-old mice of the relevant genotypes were subjected to transverse aortic constriction (TAC) or sham surgical procedure as previously described^36^. Mice were anesthetized by isoflurane inhalation to effect. Doppler echocardiography was performed on mice after TAC in order to determine pressure gradients across the aortic constriction. Mice with pressure gradients of less than 45 mmHg and fractional shortening greater than 30% at 12 weeks post-TAC were excluded from the results, as this indicated an unsuccessful surgery. Myocardial infarction (MI) was induced via permanent surgical ligation of the left coronary artery, as previously described^37^. Both sexes of mice were used for both TAC and MI surgeries. Mice were given buprenex as pain relief at a final concentration of 0.03 mg/ml by i.p. injection. Mice were then transferred to 30 °C incubators and monitored while in recovery.

### Statistics

Results are presented in all cases as mean ± SEM. Statistical analysis was performed using Prism 5 (Graphpad Software, La Jolla, CA, USA). Experiments were analyzed using Student’s t-test or one-way ANOVA with Newman-Keuls multiple comparisons test. P-values less than 0.05 were considered significant.

### Data availability

All data for this study are contained within the paper as primary figures or supplemental figures or supplemental tables. No additional data discussed herein are contained elsewhere.

## Electronic supplementary material

Supplementary Information 
