# Supplementary material for: Mitsugumin 29 regulates t-tubule architecture in the failing heart
Source: Sci Rep. 2017 Jul 13;7:5328. doi: 10.1038/s41598-017-05284-2 (PMC5509714; doi:10.1038/s41598-017-05284-2)
Supplement: Supplementary file 1 — Supplementary Information [file 41598_2017_5284_MOESM1_ESM.pdf]

SUPPLEMENTARY FIGURES AND TABLES

Mitsugumin 29 regulates t-tubule architecture in the failing heart

Robert N. Correll[1], Jeffrey M. Lynch[1], Tobias G. Schips[1], Vikram Prasad[1], Allen J. York[1], Michelle A. Sargent[1], Didier X.P. Brochet[2], Jianjie Ma[3], Jeffery D. Molkentin[1,4,*]

[1] Department of Pediatrics, University of Cincinnati, Cincinnati Children's Hospital Medical Center, Cincinnati, Ohio, 45229 USA
[2] Department of Physiology, University of Maryland School of Medicine, Baltimore, Maryland, 21201 USA
[3] Department of Surgery, The Ohio State University, Columbus, Ohio, 43210 USA
[4] Howard Hughes Medical Institute, Cincinnati, Ohio, 45229 USA
[*] Corresponding author: jeff.molkentin@cchmc.org

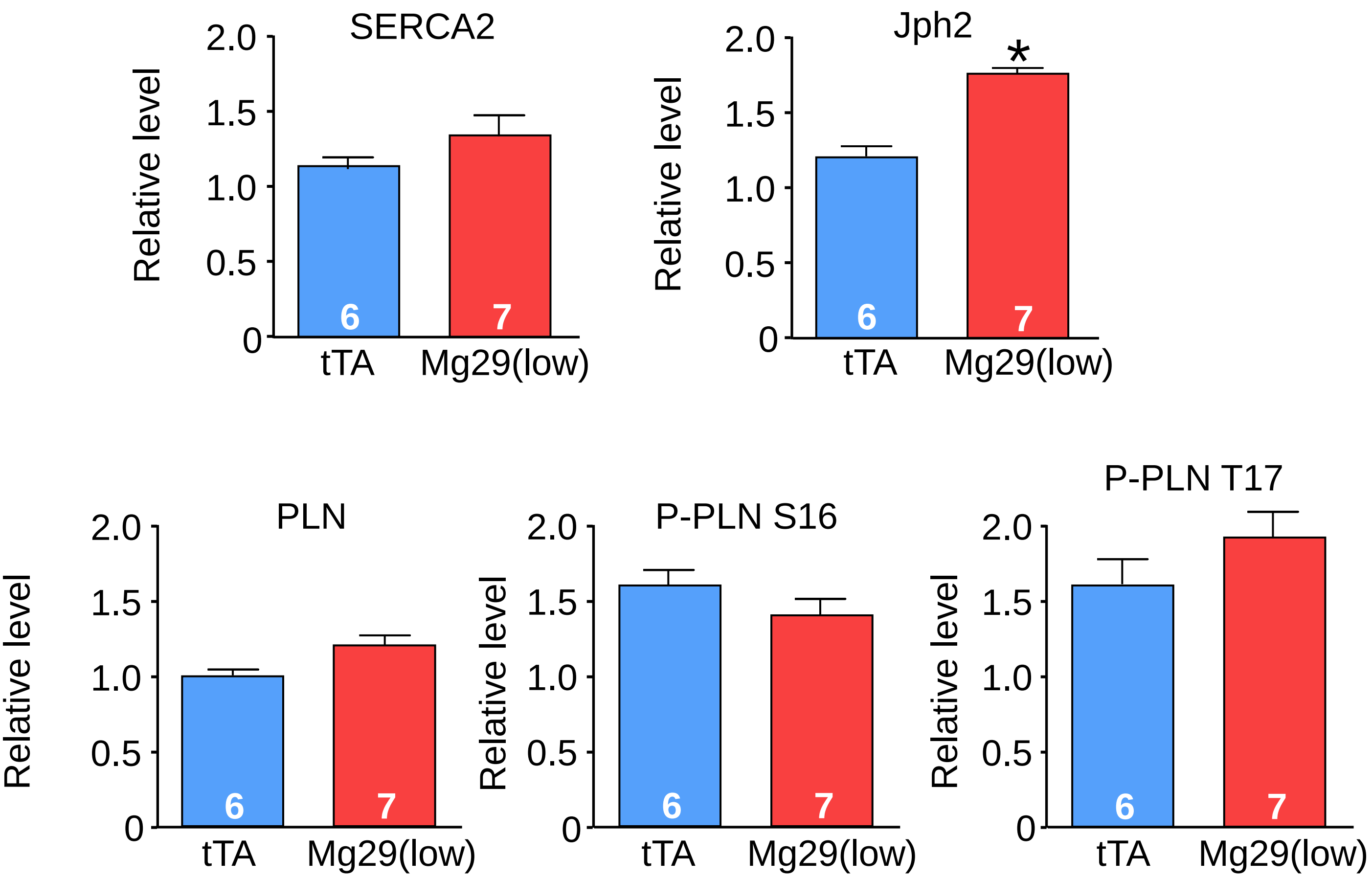

Supplementary Figure S1. Quantification of immunoblots from Figure 1d reveals no significant changes in protein expression of Ca^{2+} handling proteins SERCA2, or PLN, although there was a mild increase in Jph2 expression. Phosphorylation of PLN at S16 or T17 was also not significantly altered.

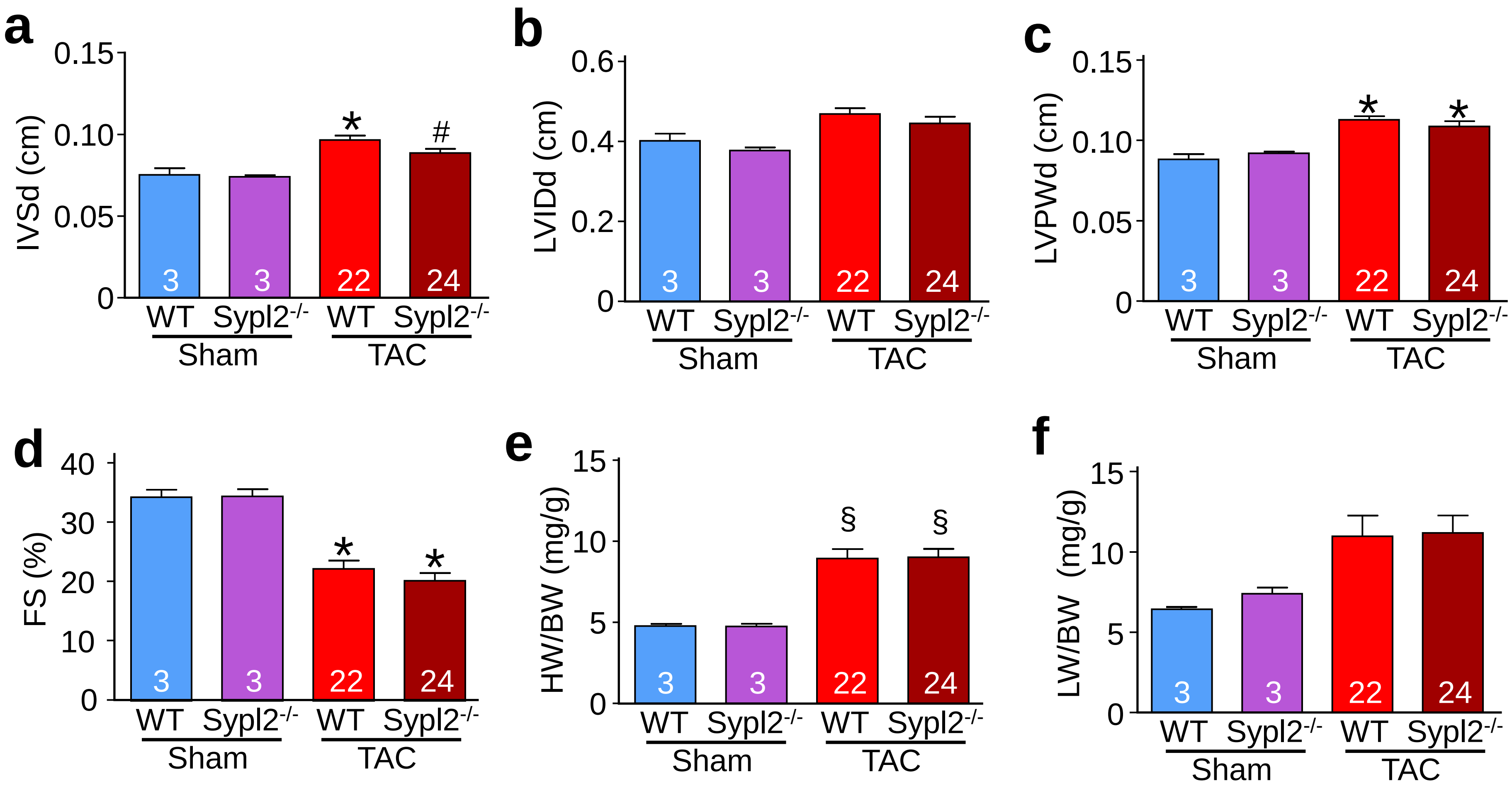

Figure S2. Loss of Mg29 in *Sypl2* gene-deleted mice has no effect on disease progression after 12 weeks of long-term pressure overload. (**a**) Intraventricular septum in diastole (IVSd), (**b**) left ventricular chamber dimension in diastole (LVIDd), (**c**) left ventricular posterior wall thickness in diastole (LVPWd), (**d**) fractional shortening percentage (FS%) all measured by echocardiography (**e**) Heart weight normalized to body weight (HW/BW) and (**f**) lung weight normalized to body weight (LW/BW). Number of mice analyzed is shown in the graphs. *P<0.05 versus sham surgery of the same genotype. $^{\#}$P<0.05 versus tTA of same surgical group. §P<0.05 versus sham control by t-test, although by ANOVA the data were not quite significant as an entire group.

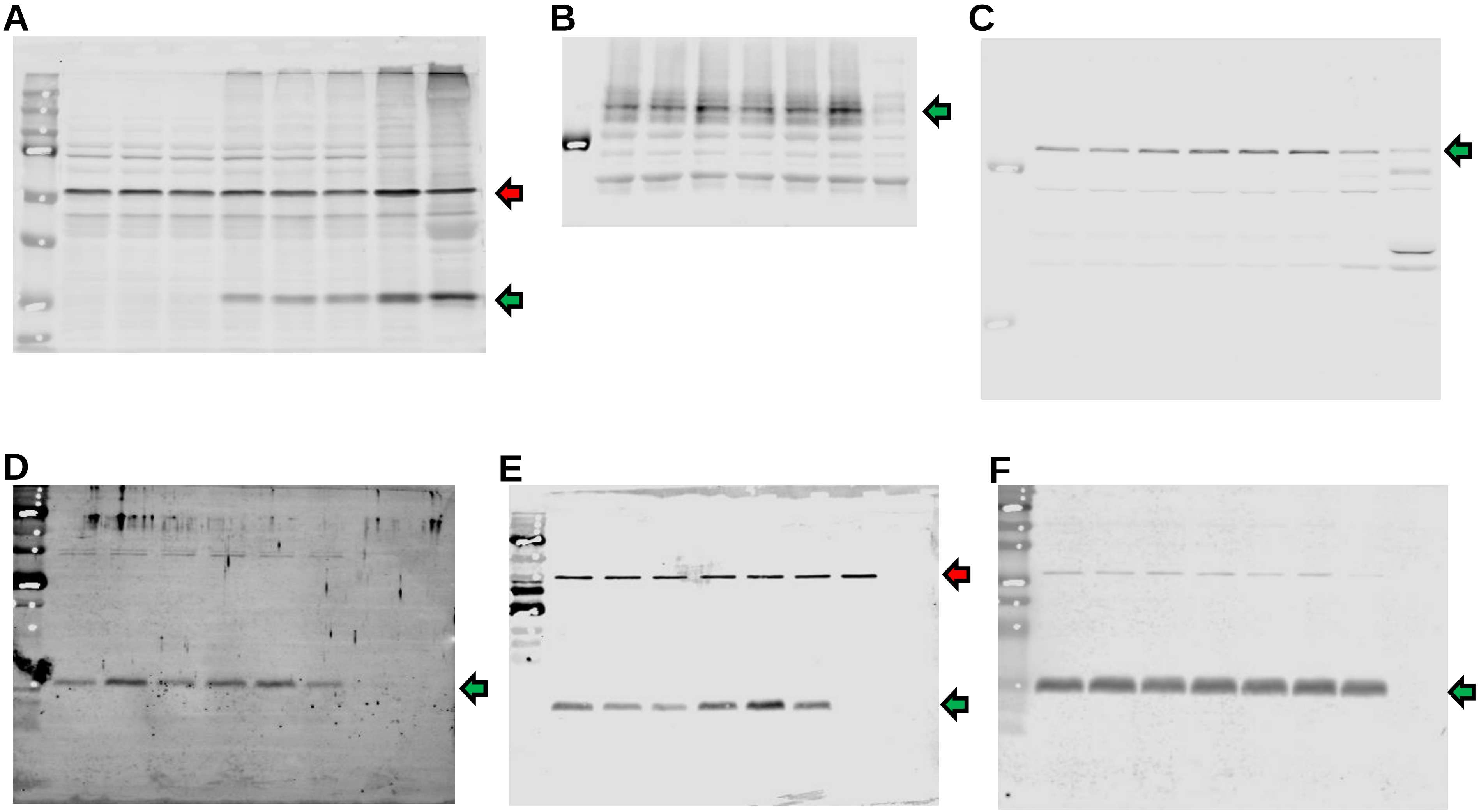

Figure S3. Uncropped images of immunoblots from Fig. 1. (A) Immunoblot of β-tubulin (red arrow) and Mg29 (green arrow) from Fig. 1C. (B) Immunoblot of SERCA2 (green arrow) from Fig. 1D. (C) Immunoblot of Jph2 (green arrow) from Fig. 1D. (D) Immunoblot of p-PLN S16 (green arrow) from Fig. 1D. (E) Immunoblot of GAPDH (red arrow) and p-PLN T17 (green arrow) from Fig. 1D. (F) Immunoblot of total PLN (green arrow) from Fig. 1D.

Supplementary Table S1. Echocardiographic parameters from Mg29(Low) TG cross with Csrp3 gene-deleted mice.

		LVIDd	LVPWd	IVSd	LVIDs	LVPWs	IVSs	%FS	N
**WT**	**tTA**	0.4097 ± 0.01424	0.0980 ± 0.009292	0.07167 ± 0.009207	0.2713 ± 0.004910	0.1297 ± 0.008667	0.1103 ± 0.005925	33.67 ± 1.510	3
	**Mg29**	0.3925 ± 0.006985	0.0980 ± 0.005170	0.07233 ± 0.001667	0.2612 ± 0.008897	0.1198 ± 0.006019	0.1163 ± 0.002261	33.53 ± 1.168	6
**Csrp3-/-**	**tTA**	0.3934 ± 0.007149	0.1041 ± 0.001743	0.08132 ± 0.001420	0.2801 ± 0.007036	0.1240 ± 0.002800	0.1186 ± 0.002369	28.77 ± 1.318	25
	**Mg29**	0.3994 ± 0.007818	0.1020 ± 0.001956	0.08091 ± 0.001774	0.2664 ± 0.007493	0.1276 ± 0.002494	0.1218 ± 0.002160	33.30 ± 1.319	22

Echocardiographic measurements of diastolic left ventricle chamber internal dimensions (LVIDd), diastolic left ventricle posterior wall thickness (LVPWd), diastolic intraventricular septum thickness (IVSd), systolic left ventricle chamber internal dimensions (LVIDs), systolic left ventricle posterior wall thickness (LVPWs), systolic intraventricular septum thickness (IVSs), and fractional shortening (FS) percentage from Mg29(Low) TG or tTA mice crossed into the *Csrp3*$^{-/-}$ background, expanded from data shown in Figure 3.
*P<0.05 versus tTA (control) group. $^{\#}$P<0.05 versus Mg29 DTG group. §P<0.05 versus *Csrp3*$^{-/-}$, tTA group.

Supplementary Table S2. Gravimetric parameters from Mg29(Low) TG cross with Csrp3 gene-deleted mice.

		HW	LW	BW	HW/BW	LW/BW	N
**WT**	**tTA**	136.0 ± 10.54	189.3 ± 13.64	29.00 ± 1.922	4.683 ± 0.05452	6.529 ± 0.1780	3
	**Mg29**	124.7 ± 9.828	175.2 ± 5.512	27.68 ± 1.314	4.519 ± 0.3218	6.355 ± 0.1289	6
**Csrp3-/-**	**tTA**	154.5 ± 4.242	171.1 ± 3.543	25.68 ± 0.5499	6.022 ± 0.1219$^{*\#}$	6.691 ± 0.1283	24
	**Mg29**	151.6 ± 3.592	182.2 ± 3.152	26.57 ± 0.8622	5.748 ± 0.1065$^{*\#}$	6.940 ± 0.1593	20

Gravimetric measurements of heart weight (HW), lung weight (LW), body weight (BW), heart weight normalized to body weight (HW/BW) and lung weight normalized to body weight (LW/BW) from Mg29(Low) TG or tTA mice crossed into the *Csrp3*$^{-/-}$ background, expanded from data shown in Fig. 3. Un-normalized HW and LW were not statistically analyzed. *P<0.05 versus tTA (control) group. $^{\#}$P<0.05 versus Mg29 DTG group. §P<0.05 versus *Csrp3*$^{-/-}$, tTA group.

Supplementary Table S3. Echocardiographic parameters from Mg29(Low) TG mice or tTA control mice after 12 weeks of TAC or sham surgery.

		LVIDd	LVPWd	IVSd	LVIDs	LVPWs	IVSs	%FS	N
Sham	tTA	0.4167 ± 0.01234	0.08667 ± 0.003685	0.07250 ± 0.003263	0.2805 ± 0.01087	0.1197 ± 0.004631	0.1187 ± 0.005110	32.73 ± 0.7749	6
	Mg29	0.4068 ± 0.01775	0.09125 ± 0.006787	0.07175 ± 0.005963	0.2733 ± 0.01843	0.1148 ± 0.003660	0.1108 ± 0.007192	33.05 ± 1.900	4
12w TAC	tTA	0.4940 ± 0.01234*	0.1069 ± 0.002395*	0.09293 ± 0.002798*	0.4023 ± 0.01466*	0.1285 ± 0.003145	0.1273 ± 0.003407	19.17 ± 1.000*	30
	Mg29	0.4885 ± 0.008424*	0.1094 ± 0.001949*	0.09332 ± 0.001816*	0.3927 ± 0.009600*	0.1306 ± 0.002304	0.1327 ± 0.002909	19.88 ± 0.7968*	37

Echocardiographic measurements of diastolic left ventricle chamber internal dimensions (LVIDd), diastolic left ventricle posterior wall thickness (LVPWd), diastolic intraventricular septum thickness (IVSd), systolic left ventricle chamber internal dimensions (LVIDs), systolic left ventricle posterior wall thickness (LVPWs), systolic intraventricular septum thickness (IVSs), and fractional shortening (FS) percentage from Mg29(Low) TG or tTA mice after 12 weeks of TAC or sham surgery, expanded from data shown in Fig. 4.

*P<0.05 versus sham surgery of the same genotype. #P<0.05 versus tTA of same surgical group.

Supplementary Table S4. Gravimetric parameters from Mg29(Low) TG mice or tTA control mice after 12 weeks of TAC or sham surgery.

		HW	LW	BW	HW/BW	LW/BW	N
Sham	tTA	147.4 ± 8.960	201.7 ± 19.06	30.32 ± 3.129	4.997 ± 0.3912	6.734 ± 0.4177	5
	Mg29	140.0 ± 8.845	211.3 ± 8.648	28.93 ± 2.476	4.879 ± 0.2056	7.410 ± 0.4566	4
12w TAC	tTA	290.7 ± 12.01	312.1 ± 27.10	28.22 ± 1.171	10.56 ± 0.5101*	11.55 ± 1.112	26
	Mg29	265.6 ± 7.578	360.5 ± 21.88	25.83 ± 0.7537	10.47 ± 0.3314*	14.39 ± 0.9666*#	36

Gravimetric measurements of heart weight (HW), lung weight (LW), body weight (BW), heart weight normalized to body weight (HW/BW) and lung weight normalized to body weight (LW/BW) from Mg29(Low) TG or tTA mice after 12 weeks of TAC or sham surgery, expanded from data shown in Fig. 4. Un-normalized HW and LW were not statistically analyzed.

*P<0.05 versus sham surgery of the same genotype. #P<0.05 versus tTA of same surgical group.

Supplementary Table S5. Echocardiographic parameters from *Csrp3*$^{-/-}$, *Csrp3*$^{-/-}$ *Sypl2*$^{-/-}$ and WT control mice.

	LVIDd	LVPWd	IVSd	LVIDs	LVPWs	IVSs	%FS	N
**WT**	0.3723 ± 0.007498	0.0865 ± 0.001981	0.0655 ± 0.002148	0.2591 ± 0.006984	0.1063 ± 0.003125	0.1055 ± 0.002911	30.52 ± 1.013	18
**Csrp3-/-**	0.4253 ± 0.007327*	0.08897 ± 0.002072	0.06790 ± 0.001573	0.3128 ± 0.009414*	0.1059 ± 0.002273	0.1121 ± 0.002005	26.84 ± 1.223	29
**Sypl2-/- Csrp3-/-**	0.4314 ± 0.01350*	0.0828 ± 0.002947	0.0623 ± 0.001967	0.3409 ± 0.02145*	0.0966 ± 0.004470	0.0937 ± 0.004169*#	21.61 ± 2.737*#	10

Echocardiographic measurements of diastolic left ventricle chamber internal dimensions (LVIDd), diastolic left ventricle posterior wall thickness (LVPWd), diastolic intraventricular septum thickness (IVSd), systolic left ventricle chamber internal dimensions (LVIDs), systolic left ventricle posterior wall thickness (LVPWs), systolic intraventricular septum thickness (IVSs), and fractional shortening (FS) percentage from WT, *Csrp3*$^{-/-}$, or *Sypl2*$^{-/-}$ and *Csrp3*$^{-/-}$ mice, expanded from data shown in Figure 5.
*P<0.05 versus WT (control) group. #P<0.05 versus *Csrp3*$^{-/-}$ group.

Supplementary Table S6. Gravimetric parameters from *Csrp3*$^{-/-}$, *Csrp3*$^{-/-}$ *Sypl2*$^{-/-}$ and WT control mice.

	HW	LW	BW	HW/BW	LW/BW	N
**WT**	100.5 ± 4.314	160.4 ± 12.17	18.86 ± 0.5830	5.322 ± 0.1366	8.455 ± 0.5260	18
**Csrp3-/-**	142.8 ± 4.795	148.7 ± 2.682	20.66 ± 0.7082	6.932 ± 0.1155*	7.290 ± 0.2015	16
**Sypl2-/- Csrp3-/-**	121.4 ± 10.00	129.4 ± 10.93	16.12 ± 1.491*#	7.587 ± 0.2512*#	8.079 ± 0.3324	6

Gravimetric measurements of heart weight (HW), lung weight (LW), body weight (BW), heart weight normalized to body weight (HW/BW) and lung weight normalized to body weight (LW/BW) from WT, *Csrp3*$^{-/-}$, or *Sypl2*$^{-/-}$ and *Csrp3*$^{-/-}$ mice, expanded from data shown in Figure 5. Un-normalized HW and LW were not statistically analyzed.
*P<0.05 versus WT (control) group. #P<0.05 versus *Csrp3*$^{-/-}$ group.
